# Soil Microbiomes in Apple Orchards Are Influenced by the Type of Agricultural Management but Never Match the Complexity and Connectivity of a Semi-natural Benchmark

**DOI:** 10.3389/fmicb.2022.830668

**Published:** 2022-02-16

**Authors:** Eva Hulsmans, Gerrit Peeters, Olivier Honnay

**Affiliations:** Plant Conservation and Population Biology, Department of Biology, KU Leuven, Leuven, Belgium

**Keywords:** apple orchards, soil microbiome, agricultural management, semi-natural reference, organic agriculture, integrated pest management

## Abstract

Conversion of natural ecosystems into agricultural land may strongly affect the soil microbiome and the functioning of the soil ecosystem. Alternative farming systems, such as organic farming, have therefore been advocated to reduce this impact, yet the outcomes of different agricultural management regimes often remain ambiguous and their evaluations mostly lack a proper more natural benchmark. We used high-throughput amplicon sequencing, linear models, redundancy analyses, and co-occurrence network analyses to investigate the effect of organic and integrated pest management (IPM) on soil fungal and bacterial communities in both the crop and drive rows of apple orchards in Belgium, and we included semi-natural grasslands as a benchmark. Fungi were strongly influenced by agricultural management, with lower diversity indices and distinct communities in IPM compared to organic orchards, whereas IPM orchards had a higher AMF abundance and the most complex and connected fungal communities. Bacterial diversity indices, community composition, and functional groups were less affected by management, with only a higher network connectivity and abundance of keystone taxa in organic drive rows. On the other hand, none of the agricultural soil microbiomes matched the complexity and connectedness of our semi-natural benchmark, demonstrating that even more nature-friendly agricultural management practices strongly affect the soil microbiome and highlighting the essential role of (semi-)natural systems as a harbor of robust and functionally diverse fungal and bacterial communities.

## Introduction

Soils and their associated biodiversity play a crucial role in providing a wide range of ecosystem services in terrestrial ecosystems (Barrios, 2007; De Vries et al., 2013; van der Heijden and Wagg, 2013; Bardgett and Van Der Putten, 2014). Bacteria and fungi are essential parts of soil biodiversity, mediating fundamental ecological functions, such as nutrient cycling and carbon sequestration (Graham et al., 2016; Paul, 2016; Steffan and Dharampal, 2019), and directly influencing plant health through symbiotic and pathogenic interactions (Van Der Heijden et al., 2008; De Coninck et al., 2015). Ongoing global conversion of natural ecosystems into agricultural land poses a continuing threat to soil biodiversity and soil functioning (Orgiazzi et al., 2016), and due to the still increasing demand for food, feed, fiber, and bioenergy crops, millions of square kilometers of natural ecosystems are threatened by the expansion of agricultural land (Tilman et al., 2017; Williams et al., 2020). Soil biodiversity in agricultural lands is known to be continuously exposed to a variety of threats, including pesticide and fertilizer applications and frequent soil disturbance (Orgiazzi et al., 2016). Evidence is increasing that this results in an agricultural soil microbiome that is significantly less complex and highly different from that of more natural land uses (Creamer et al., 2016; Lammel et al., 2021).

In an effort to reduce the impact of agriculture on the environment and biodiversity, alternative farming systems, such as organic farming, are commonly advocated. Organic practices exclude the use of synthetic pesticides and fertilizers and rely more on natural processes for biological pest control and nutrient supply (Reganold and Wachter, 2016). Globally, organic farming is a booming sector with 72.3 million ha of organic farmland as of 2019, increasing more than 5-fold since 1999 (Schlatter et al., 2021). This not only indicates the need for a thorough comparison of soil microbiomes between organic and conventional farming systems, but also between farming systems and a benchmark of natural or semi-natural land. Organic farms generally harbor distinct microbial communities compared to conventional farms (Hartmann et al., 2015; Wang et al., 2016; Lupatini et al., 2017; Jurburg et al., 2020), although some studies did not find differences in bacterial community composition (Pershina et al., 2015; Hendgen et al., 2018). Soil microbiomes in organic farming systems have also been shown to have a higher total microbial biomass and activity compared to conventional systems (Esperschütz et al., 2007; Reilly et al., 2013; Lori et al., 2017; Martínez-García et al., 2018). Additionally, a higher microbial diversity has been found under organic management (Tuck et al., 2014; Hartmann et al., 2015). On the other hand, Jurburg et al. (2020) did not find differences in microbial richness between conventional and organic farms, while others found inconsistent responses of fungal and bacterial diversity (Hendgen et al., 2018; Lupatini et al., 2018; Finn et al., 2021).

Overall, the response of soil communities to agricultural management is complicated and cannot be captured by simple biodiversity indices (Hartmann et al., 2015). Due to their essential importance for ecosystem functioning, changes in the community composition and complexity of soil microbiomes can be expected to be more important than in species diversity alone (Wagg et al., 2019). The composition of functional groups is known to strongly impact soil functioning through the regulation of key ecosystem processes (Barrios, 2007). Arbuscular mycorrhizal fungi (AMF) for instance form a mutualistic relationship with a wide range of plant species and allocate nutrients to the plant in exchange for photosynthates (Van Der Heijden et al., 2008; Powell and Rillig, 2018). In addition, AMF can alter soil abiotic conditions and other plant biotic interactions, further influencing plant production and ecosystem functioning (Chaudhary, 2016; Powell and Rillig, 2018). Microbiomes are highly structured communities and form complex networks where microorganisms associate either directly or indirectly, in turn affecting functioning (Fuhrman, 2009; Banerjee et al., 2016). Creating co-occurrence networks can help elucidate these complex interactions and, together with network analysis, it allows both the quantification of association shifts in different environmental settings (Barberán et al., 2012; Layeghifard et al., 2017; de Vries et al., 2018) and the identification of keystone taxa (Herren and McMahon, 2018). A higher network complexity is expected to result in more resilient microbial communities, due to complementation and redundancy effects, and has been found to positively influence nutrient cycling (Wagg et al., 2019). Agricultural intensification has been shown to lower root fungal network complexity and the number of keystone taxa (Banerjee et al., 2019), whereas abandoned arable fields harbor more connected networks as ecological restoration progresses, improving carbon uptake (Morriën et al., 2017). Throughout the succession toward more natural ecosystems, soil microbiomes generally become more complex and stable and contain less soil pathogens and more mycorrhizal fungi (Kardol et al., 2006; Walker et al., 2010; Wagg et al., 2020).

So far, most research investigating effects of agricultural practices on soil microbial communities has been performed in arable systems, whereas perennial crop systems, such as orchards, have received less attention. Studies in orchards have mostly focused on a limited number of taxa, often AMF, and to our knowledge, the microbial network structure under different orchard management practices has not yet been investigated (Radić et al., 2014; Meyer et al., 2015; Meng et al., 2016; Van Geel et al., 2017). Furthermore, in order to be able to fully appreciate the effects of different agricultural management strategies on the soil microbiome and to quantify to what extent microbial communities have been degraded as a result of agricultural practices, it is highly informative to include a natural or semi-natural benchmark in the comparison (Katayama et al., 2019).

In this study, we used high-throughput amplicon sequencing to characterize soil fungal and bacterial communities in 30 Belgian apple orchards under conventional farming following Integrated Pest Management (IPM), and in 30 apple orchards under certified organic management. Furthermore, we added 30 semi-natural grasslands as a natural benchmark. Our objectives were to evaluate the effects of the agricultural management system on (i) taxon richness and diversity; (ii) phylogenetic diversity, (iii) community composition, and (iv) functional group abundance, all in comparison with the semi-natural reference system. Additionally, we aimed to (v) compare co-occurrence networks and their complexity between the management and land use types and to identify key microbial taxa within these networks.

## Materials and Methods

### Study Sites and Sampling

The sampled locations were located in Flanders, the northern region of Belgium, from 50°44’52.6” to 51°16’12.9“N and 3°39’14.2” to 5°24’47.5“E (Supplementary Figure S1). The region has a temperate oceanic climate with an average temperature of 9.8°C and an average annual precipitation of 925 mm. Soil samples were taken in October 2018 in 30 orchards managed under IPM and in 30 organically managed orchards. IPM orchards were fertilized using synthetic fertilizers at different time points during the year, whereas pests were managed using a combination of biological, mechanical, and physical methods, such as mating disruption, with chemical pesticides applied only as a last resort. All organic orchards were in agreement with European legislation [Council Regulation (EC) No 834/2007], meaning that they applied organic fertilizers and barred synthetic pesticides. Organic orchards instead also relied on non-chemical methods, such as the propagation of natural pest predators, complemented with organic pesticides, such as sulfur when necessary. All orchards contained Jonagold apple trees (*Malus domestica* “Jonagold”) at production age. In each orchard, three plots were randomly established at least 10 m from the orchard edge. In each plot, five soil samples (10 cm depth) were taken using a soil auger (2 cm diameter) from two 1 m^2^ sub-plots, one in the crop row, and one in the adjacent drive row, and pooled into one bulk sample per sub-plot. Crop rows are either sprayed with herbicides (IPM) or mechanically weeded (organic) to remove weeds, while drive rows are mown to maintain herbaceous vegetation. In addition, 30 reference semi-natural grasslands with similar soil type, hydrology, and slope were sampled in three 1 m^2^ plots in their center in a similar way. These unfertilized grasslands are managed by mowing or grazing for nature conservation purposes. This resulted in 90 soil samples from crop rows and 90 samples from drive rows in both the organic and IPM orchards (360 samples in total) and 90 samples from semi-natural grasslands. After mixing, the soil samples were divided in two parts, one part was stored at 5°C for chemical analyses, and the other part was stored at −20°C for DNA extraction.

### Soil Chemical Analyses

Soil water content was determined by drying soil at 50°C for 24 h or until a stable weight was achieved. To quantify organic matter content, the dried soil was subsequently put in a muffle furnace at 600°C to combust organic compounds. Soil pH was measured using a glass electrode in a 1:10 soil/water mixture. We determined plant-available nitrogen content in the soil, under the form of ammonium and nitrate availability, by shaking 10 g of soil in 200 ml of 1 M potassium chloride solution for 1 h. Subsequent colorimetric analysis was performed using a segmented flow auto analyzer (Skalar, Breda, the Netherlands). As a measure of plant-available phosphorus in the soil, Olson P was quantified by shaking 2 g of dried soil for 30 min with a 0.5 M sodium bicarbonate solution at pH 8.5, followed by colorimetric analysis using the molybdenum blue method (Lajtha et al., 1999).

### DNA Extraction, PCR Amplification, and High-Throughput Amplicon Sequencing

Genomic DNA was extracted from 250 mg of soil using the soil DNA Isolation Plus Kit (Norgen Biotek Crop., Thorold, ON, Canada) following the manufacturer’s instructions. The DNA extracts were diluted 10 times before PCR amplification. Fungal communities were profiled through PCR amplification targeting the ITS2 region of the rRNA gene using the sample-specific barcode-labeled versions of the primers ITS86F and ITS4 (Waud et al., 2014; dual-index sequencing strategy, Kozich et al., 2013). Bacterial communities were characterized targeting the hypervariable V4 region of the 16S rRNA gene with the 515F/806R primer pair (Caporaso et al., 2011). PCR was performed in a 25 μl volume, containing 0.25 mM of each dNTP, 0.5 μM of each primer, ×1 ALLin HiFi Buffer, 1 U ALLin HiFi DNA Polymerase (HighQu, Kraichtal, Germany), and 1 μl genomic DNA. DNA samples were denatured at 95°C for 1 min, followed by 35 cycles of 15 s at 95°C, 15 s at 60°C, and 15 s at 72°C, and finally 3 min at 72°C. Amplicons were purified using the Agencourt AMPure XP kit (Beckman Coulter Life Sciences, Indianapolis, IN, United States). Next, purified double-stranded DNA (dsDNA) amplicons were quantified using the Quant-iT PicoGreen dsDNA Assay Kit and Qubit fluorometer (Invitrogen), and pooled in equimolar quantities in four amplicon libraries. These amplicon libraries were loaded on an agarose gel, and the amplicon of the expected size was excised and purified using the QIAquick Gel Extraction Kit (Qiagen). After dilution to 2 nM, the amplicons were sequenced at the Genomics Core UZ Leuven on an Illumina Miseq platform using 2 × 250 paired-end Illumina chemistry with v2 500 cycle reagent kit (Illumina, San Diego, CA, United States).

### Bioinformatics

Quality-filtered sequences were clustered into operational taxonomical units (OTUs) following the recommended pipeline in USEARCH (v. 11; Edgar, 2013). First, paired-end reads from the obtained demultiplexed FASTQ files were merged to create consensus sequences (fastq_mergepairs). Next, reads were quality filtered (fastq_filter), allowing a maximum expected error of 0.5 for the individual sequences. Sequences were then dereplicated and sorted by abundance (fastx_uniques). To improve the accuracy of diversity estimates, singletons were removed before clustering (Brown et al., 2015). Finally, sequences were clustered into OTUs defined at 97% sequence similarity (cluster_otus), during which chimeric sequences were also removed (Edgar, 2013).

### Data Analyses

All analyses were performed using R (R Core Team, 2021, v. 4.0.0) and separately for the fungal and the bacterial datasets. More than 20,100 fungal and 36,000 bacterial effective sequences were obtained on average for each sample. Sufficient sequencing depth was assessed and confirmed for all samples through rarefaction using the vegan package (Oksanen et al., 2020). To avoid bias due to differences in sequencing depth, reads were randomly resampled from each sample to an equal number of reads (10.000 for fungi and 20.000 for bacteria). Statistical analyses were done in two steps. First, all data from the orchards were compiled in order to assess the effects of orchard management (IPM vs. organic) and location (crop row vs. drive row), further referred to as management models. Second, to model the effects of land use (IPM vs. organic. vs. semi-natural grassland) on the microbiome, the data from the herbaceous drive rows of both orchard types and the data from the reference semi-natural grasslands were used. We omitted the crop rows from this analysis in order to obtain a more conservative estimate of the effects of agricultural management as compared to the semi-natural benchmark. These models are further referred to as land use models. A set of covariates, i.e., soil characteristics (water content, organic matter, pH, ammonium, nitrate, and P) and spatial predictors (distance-based Moran’s eigenvector maps, dbMEMs) were added to all models. dbMEMs were included to account for possible effects from the spatial structure of the study sites (Legendre and Legendre, 2012).

Operational taxonomical unit taxonomic diversity was quantified by applying the Hill number framework, using both diversity of order 0 (OTU richness) and order 1 (^1^D; Hill, 1973), and phylogenetic diversity (PD) was calculated using Faith’s PD (Faith, 1992). A generalized linear mixed model (GLMM) following a Poisson distribution and log link function was used to test effects of land use and management and the variables described above on OTU richness, while linear mixed models (LMM) were used to test effects on ^1^D and PD. To assess effects on functional groups, we assigned functionality using the FUNGuild for fungal OTUs (Nguyen et al., 2016) and METAGENASSIST for bacterial OTUs (Arndt et al., 2012). The read count of fungal saprotrophs, AMF, and plant pathogens as well as the four most dominant metabolic groups of bacteria (sulfite reducer, ammonia oxidizer, dehalogenation, and xylan degrader) were used as response variables in individual GLMMs following a Poisson distribution and log link function. In all management models, sub-plot was nested within plot as a random factor, whereas plot was included as a random factor in all land use models. Model selection was based on the Akaike’s information criterion (AIC) with a backward selection procedure. Variance inflation factors (VIFs) were calculated to detect multicollinearity between the explanatory variables and were low (all <3). Next, we explored variation in fungal and bacterial community composition through canonical redundancy analyses (RDA), using the vegan package (Oksanen et al., 2020) with management and location (management models) or land use (land use models), soil characteristics, and dbMEM variables as explanatory variables. Forward model selection was done with 999 permutations.

To identify specific OTUs that were associated with a specific land use and location, we performed correlation-based association analyses using the multipatt function of the indicspecies package (De Caceres et al., 2016). For visualization, all OTUs with significant associations (*p* < 0.05) were displayed as target nodes in a network with the five land use and location types as source nodes. Networks were visualized using the spring-embedded layout algorithm in Cytoscape (Shannon et al., 2003). We then investigated co-occurrence patterns by constructing networks for each land use type separately. Interactions within the networks consisted of positive and significant Spearman’s correlations (*ρ* > 0.6 and *p* < 0.05). All network topology parameters were calculated using the igraph package in R (Csardi and Nepusz, 2006), whereas the networks were visualized in Gephi (Bastian et al., 2009). The nodes, corresponding with OTUs, are the fundamental units in these networks, while the edges form the connections between these nodes. The degree of a node then represents the total number of edges associated with that node. Network diameter is the largest distance between two nodes in a network, and average path length is the mean of the shortest distance between all pairs of network nodes in the network. Closeness centrality quantifies the inverse distance to all other nodes in the network and reveals the importance of nodes for the dissemination of information. Betweenness centrality on the other hand indicates the level in which nodes act as a bridge connecting different clusters of the graph. Modularity expresses the strength of division within a network into clusters. The OTUs with the highest degree and highest closeness centrality and the lowest betweenness centrality were identified as keystone taxa (Berry and Widder, 2014). OTUs with degree > 5, closeness centrality > 0.005, and betweenness centrality < 40 were selected as keystone taxa for the fungal networks, and OTUs with degree > 15, closeness centrality > 0.03, and betweenness centrality < 50 for the bacterial networks.

## Results

### Diversity Indices

**Figure 1:**
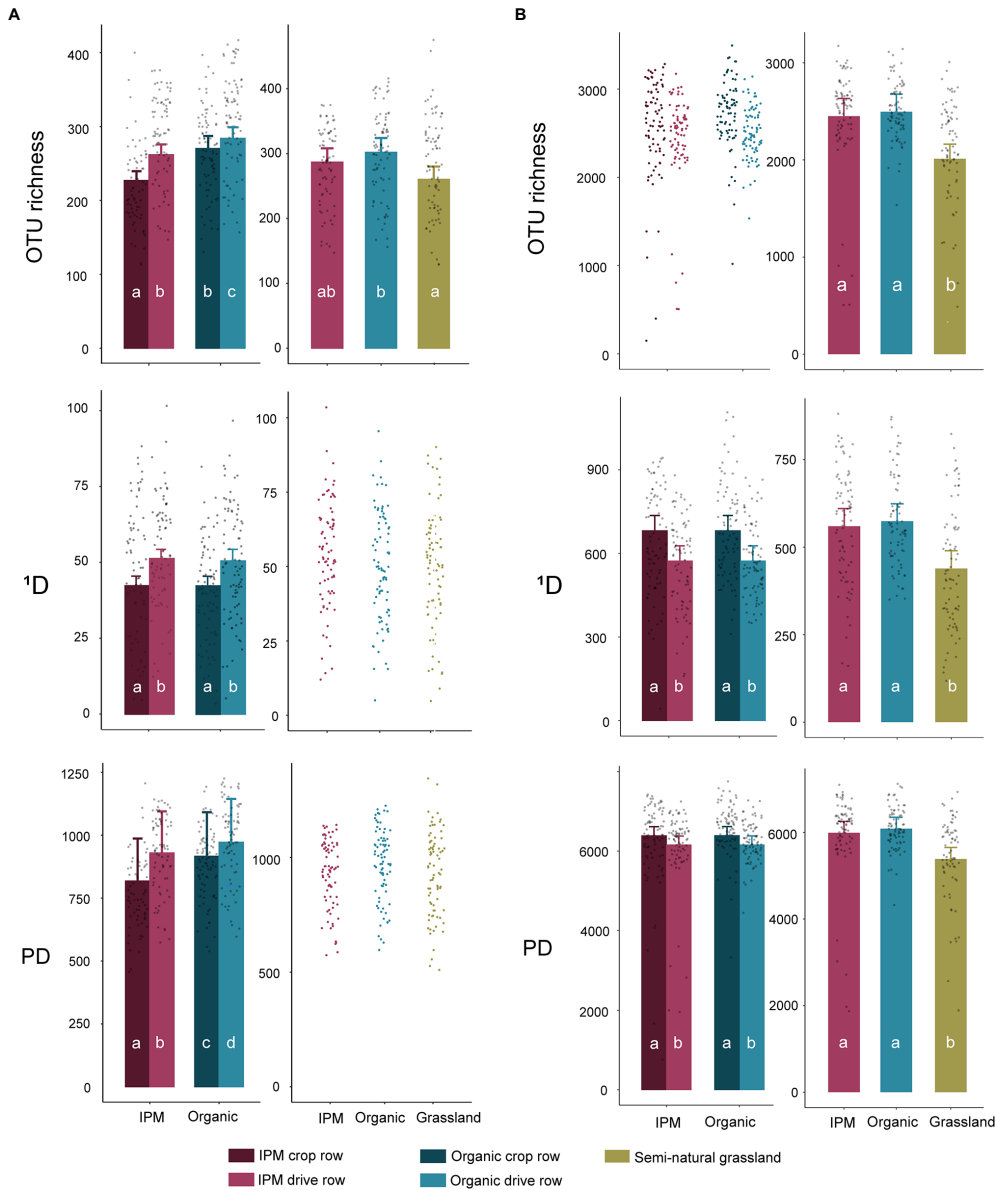
Diversity indices in integrated pest management (IPM) and organic orchards with a semi-natural grassland reference. Modeled mean values ±1SE and raw data points of Hill numbers of order 0 [operational taxonomical unit (OTU) richness] and order 1 (^1^D) and phylogenetic diversity (PD) for fungal **(A)** and bacterial **(B)** communities across land use types. When land use or location was not selected during the model selection procedure, only raw data points are shown. Left panels: crop rows (dark) and drive rows (light) of IPM and organic orchards. Right panels: drive rows of IPM and organic orchards with a semi-natural grassland reference. Same lowercase letter indicates no statistically significant (*p* < 0.05) difference between land uses.

In the management model, fungal OTU richness was significantly affected by management (*p* = 0.0004), location (*p* < 0.0001), and by their interaction (*p* < 0.0001), with a higher richness in organic orchards and in drive rows. The difference between crop row and drive row was smaller in organic orchards (Figure 1A; Supplementary Table S1). In contrast, fungal ^1^D was only affected by location in the management model (*p* < 0.0001), with a higher ^1^D in drive rows than in crop rows (Figure 1A; Supplementary Table S1). Fungal PD was significantly higher in organic orchards (*p* = 0.0084) and in drive rows (*p* = 0.0001) in the management model. Bacterial OTU richness was not significantly affected by management or location in the management model, but bacterial ^1^D and PD were significantly higher in crop rows compared to drive rows (both *p* < 0.0001; Figure 1B; Supplementary Table S1). In the land use models, fungal OTU richness was significantly lower in grasslands compared to organic orchards (*p* = 0.015), whereas grasslands and organic orchards both did not significantly differ in fungal OTU richness compared to IPM orchards (Figure 1A; Supplementary Table S1). Fungal ^1^D and PD were unaffected by land use (Figure 1A; Supplementary Table S1). All bacterial diversity indices were significantly lower in grasslands compared to both orchard types (*p* < 0.0001, *p* = 0.0003, and *p* = 0.0004 for OTU richness, ^1^D, and PD, respectively; Figure 1B; Supplementary Table S1). Overall, the significant differences that were found between management types for the studied diversity indices in the management models disappeared in the land use models.

### Community Composition

**Figure 2:**
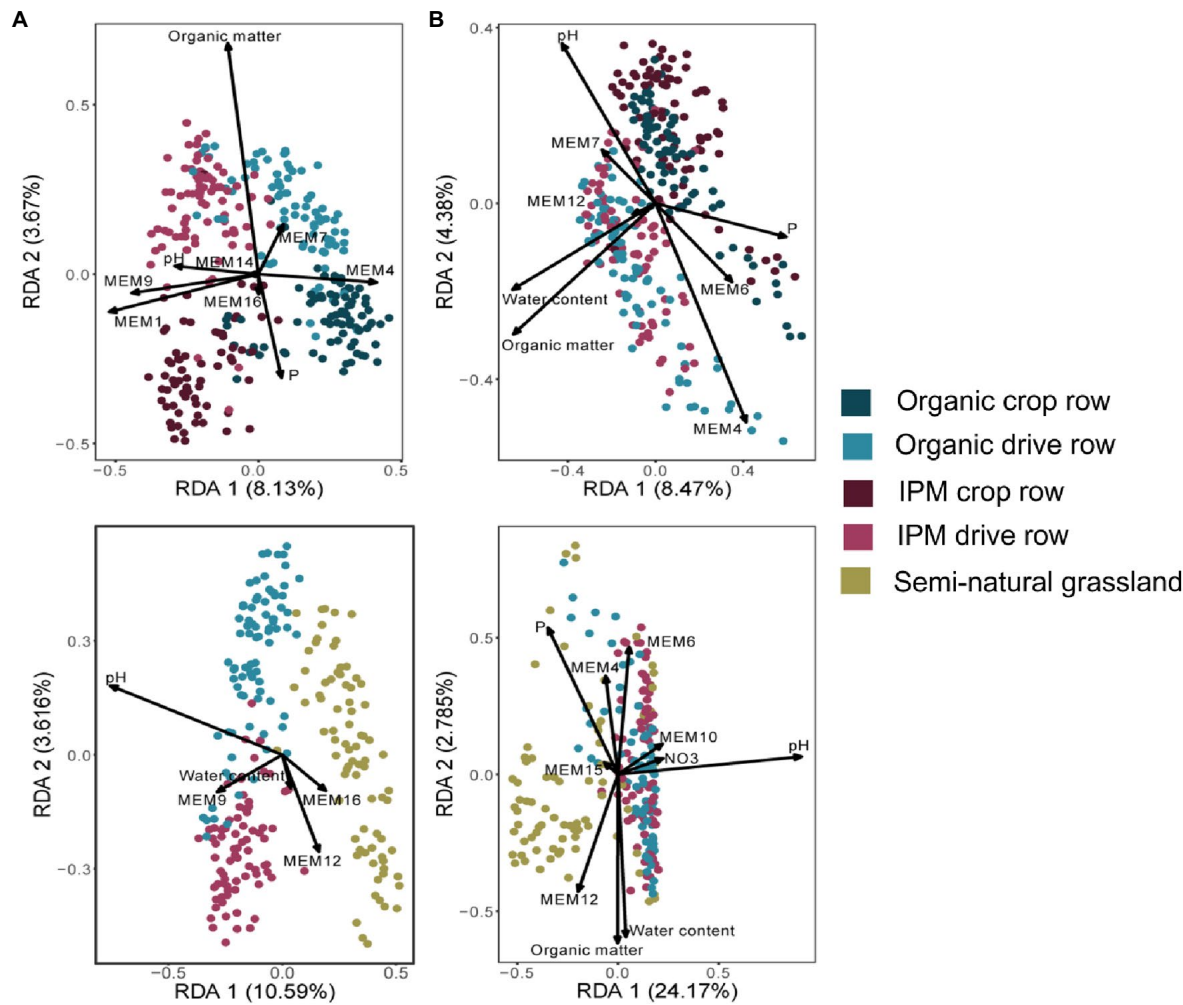
Redundancy analysis (RDA) ordination biplots of communities in IPM and organic orchards with a semi-natural grassland reference. RDA biplots generated of fungal **(A)** and bacterial **(B)** communities in crop rows and drive rows of IPM and organic orchards (upper plots) and for drive rows of IPM and organic orchards with a semi-natural grassland reference (lower plots). Arrows indicate variables selected in the model selection procedure (Supplementary Table S2).

The management RDA selected management, location, and their interaction as significantly explaining variation in fungal orchard communities (Figure 2A; Supplementary Table S2). Selected covariates included phosphorus, organic matter, and soil pH, whereas spatial vectors also significantly explained community variation. The final model explained 17% of the variation. The land use RDA selected land use as a factor significantly explaining fungal community composition together with covariates phosphorus, water content, soil pH, and spatial vectors (Figure 2A; Supplementary Table S2). The final model explained 16% of the variation. Variation in bacterial community composition in orchards (land use model) was significantly affected by the factor location while phosphorus, water content, organic matter, and soil pH and spatial vectors were selected as covariates (Figure 2B; Supplementary Table S2). The final model explained 16% of the variation. In the land use RDA, land use was selected as a factor explaining variation in bacterial community composition together with covariates soil pH, phosphorus, water content, organic matter, nitrate, and spatial vectors (Figure 2B; Supplementary Table S2). The final model explained 30% of the variation.

### Functional Groups

**Figure 3:**
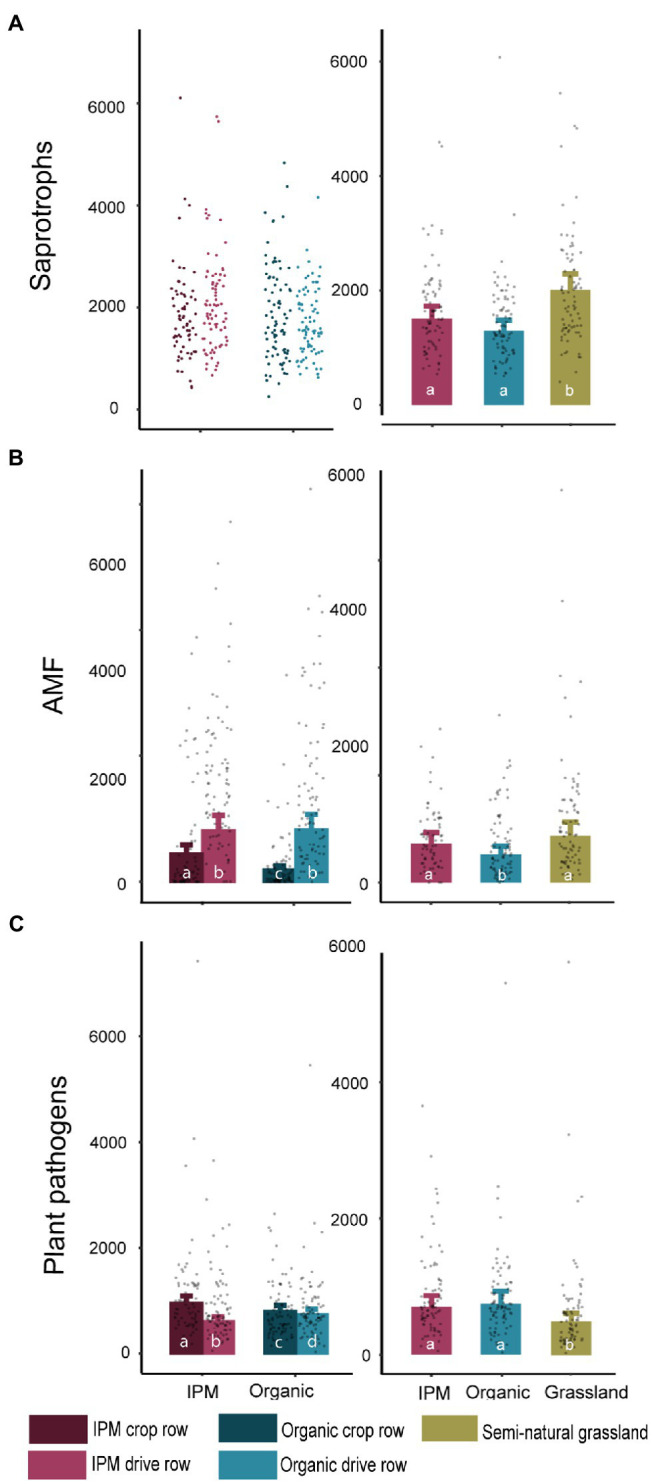
Read count of fungal functional groups in IPM and organic orchards with a semi-natural grassland reference. Modeled mean values ±1SE and raw data points of saprotrophic fungi, arbuscular mycorrhizal fungi (AMF), and plant pathogens across land use types. When land use or location was not selected during the model selection procedure, only raw data points are shown. Left panels: crop rows (dark) and drive rows (light) of IPM and organic orchards. Right panels: drive rows of IPM and organic orchards with a semi-natural grassland reference. Same lowercase letter indicates no statistically significant (*p* < 0.05) difference between land uses.

The number of saprotrophic fungal reads in the management model was not influenced by management or location (Figure 3A; Supplementary Table S3). The abundance of AMF was influenced by management (*p* = 0.045), location (*p* < 0.0001), and their interaction (*p* < 0.0001), with a higher abundance in IPM and in drive rows, though the difference between drive rows and crop rows was larger in organic orchards. The pathogen abundance was significantly affected by location (*p* < 0.0001) and the interaction between management and location (*p* < 0.0001; Figure 3; Supplementary Table S3), with more pathogen OTUs in crop rows compared to drive rows in IPM orchards. In the land use model, a significantly higher number of saprotroph reads was found in grasslands compared to both orchard types (*p* = 0.0002; Figure 3A; Supplementary Table S3). The number of AMF reads was significantly lower in organic orchards compared to IPM and grasslands (*p* = 0.024; Figure 3B; Supplementary Table S3). The number of plant pathogen reads was lower in grasslands compared to both orchard types (*p* = 0.013; Figure 3C; Supplementary Table S3). Bacterial functional groups were less affected by management and land use than fungal functional groups and only the number of ammonia oxidizer reads in the land use model was significantly affected by land use (*p* < 0.0001; Supplementary Figure S2; Supplementary Table S4).

### Indicator Taxa

**Figure 4:**
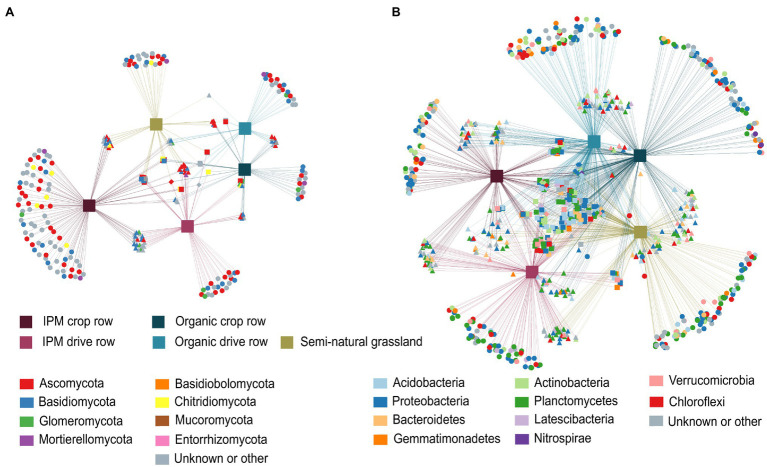
Correlation-based association networks across land use types. Networks are constructed at the OTU level of the fungal **(A)** and bacterial **(B)** communities across IPM and organic crop rows and drive rows with a semi-natural grassland reference. Only statistically significant OTU associations are displayed as source nodes (*p* < 0.05).

In the fungal network, 258 OTUs significantly associated with one or more of the three land use types. Of these OTUs, 63.9% were associated with just one land use type and almost half of these taxa (30.6%) were associated with IPM crop rows. These taxa included a higher number of Glomeromycota, in addition to mainly Ascomycota and Basidiomycota. Both IPM orchard locations combined shared more OTUs with semi-natural grasslands (17.1%) than did organic orchards (3.1%; Figure 4A). In the bacterial networks, a total of 870 OTUs were significantly associated with at least one land use type, with 42.3% of these OTUs associated with one particular type and a fairly even distribution of the number of habitat specialists and their assigned phyla across land use types. However, we found more Acidobacteria habitat specialists in organic and more Planctomycetes habitat specialists in IPM orchards. There was more shared indicator bacterial OTUs between organic orchards and grasslands (19.4%) than between IPM orchards and grasslands (7.7%; Figure 4B).

### Co-occurrence Networks

**Figure 5:**
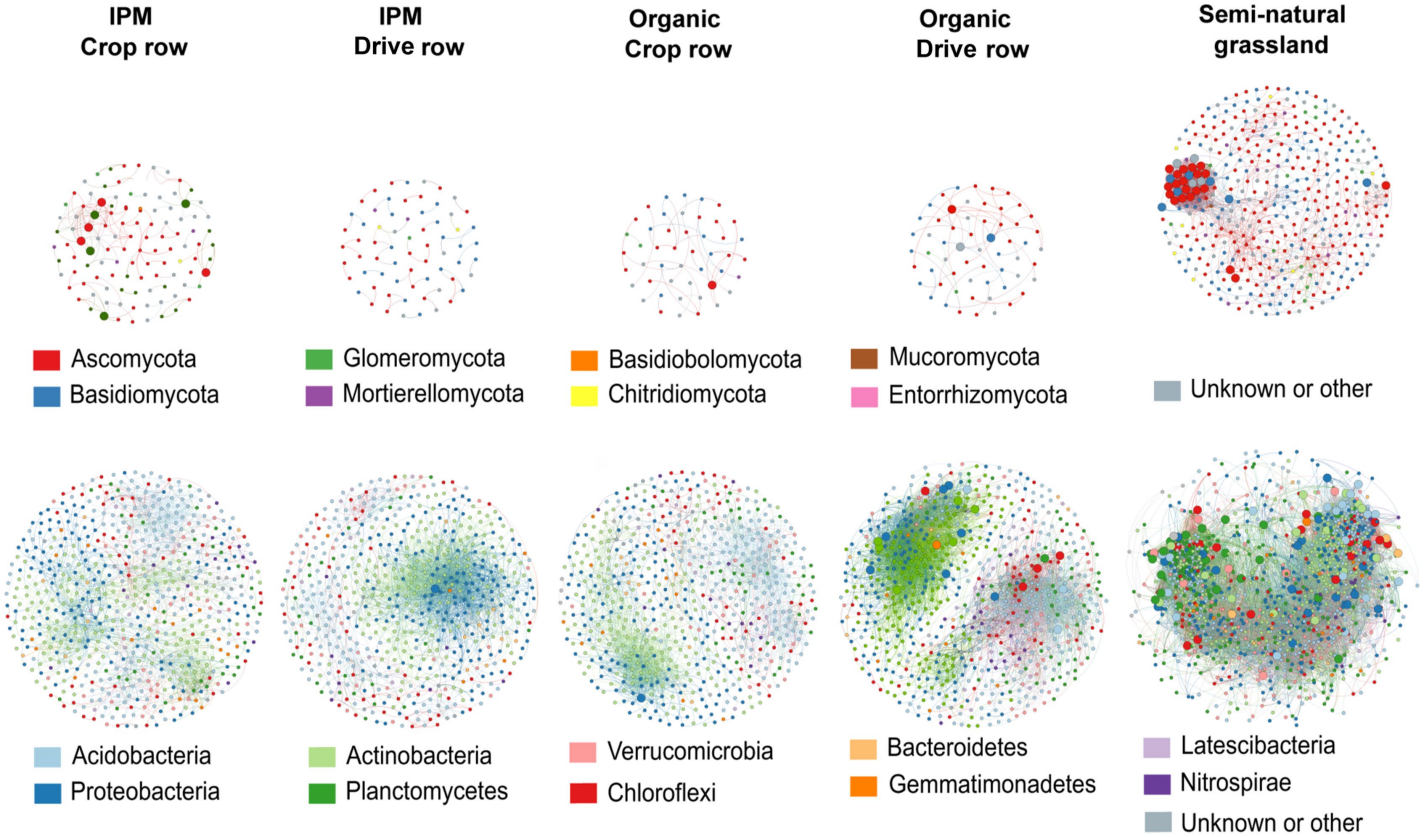
Co-occurrence networks across land use types. Networks are generated for fungal (upper plots) and bacterial (lower plots) communities across IPM and organic crop rows and drive rows with a semi-natural grassland reference. Only interactions with positive and significant Spearman’s correlations are displayed (*p* > 0.6 and *p* < 0.05), with OTUs as nodes and interactions as edges. Large nodes indicate the keystone taxa.

We separately evaluated co-occurrence networks for all land use types involved in this study, based on significant correlations, revealing notable differences in the topology between networks. Both for fungal and bacterial networks, the grassland network was the most densely populated, complex and connected compared to all orchard networks, and it contained the highest number of keystone taxa (Figure 5; Supplementary Table S5). The fungal grassland network contained 31 keystone taxa, mainly belonging to the phyla Ascomycota and Basidiomycota. Interestingly, the fungal IPM crop row network was the most complex of all fungal orchard networks (Figure 5; Supplementary Table S5). It contained eight keystone taxa belonging to the phyla Ascomycota and Glomeromycota. The other fungal networks were less complex and connected and contained less keystone taxa; the organic drive row network had three keystone taxa, while the organic crop row network had one, and the IPM drive row network contained no keystone taxa at all (Figure 5; Supplementary Table S5). Bacterial networks were more complex and contained a higher number of nodes than fungal networks. There was a high abundance of keystone taxa in the bacterial grassland network, with a total of 110 taxa belonging to a variety of phyla—mainly Chloroflexi, Planctomycetes, and Proteobacteria (Figure 5). Of the bacterial orchard networks, the organic drive row network seems to be best connected, with a degree equal to the grassland network, a relatively high closeness centrality, high betweenness centrality, and low average path length (Figure 5; Supplementary Table S5). It contained 22 keystone taxa, most belonging to Proteobacteria, Actinobacteria, and Chloroflexi. The other orchard networks showed similar topology values (Figure 5; Supplementary Table S5). The organic crop row and IPM drive row each had one keystone taxon of the phylum Proteobacteria. The bacterial IPM crop row network did not contain any keystone taxa.

## Discussion

To our knowledge, this is the first study to evaluate the effects of IPM and organic management in a perennial agricultural system on the soil microbiome in reference to a semi-natural benchmark. We generally found significant effects of orchard management on fungal diversity indices, community composition, the abundance of functional groups, and the complexity of co-occurrence networks. In contrast, whereas the complexity of bacterial networks was affected by orchard management, bacterial diversity, community composition, and the abundance of functional groups were not. On the other hand, differences in microbial diversity between the drive rows of both orchard management systems were very small and disappeared when compared to a semi-natural benchmark. None of the agricultural soil microbiomes matched the complexity and connectedness of the semi-natural benchmark.

### Management and Location Effects on Soil Fungi

Fungal communities were significantly different and OTU richness and phylogenetic diversity were higher in organic orchards compared to IPM orchards, and in drive rows compared to crop rows. However, the higher OTU richness found in organic orchards compared to IPM orchards was more noticeable in crop rows and less so in drive rows. Previous work in perennial agricultural systems did not find management effects on fungal OTU richness, yet they did find management effects on fungal community composition (Hendgen et al., 2018; Jurburg et al., 2020). An important driver may be the use of herbicides in the IPM crop rows, which prevents the emergence of herbaceous plant species and might affect plant species diversity in the adjacent drive row through spray drift as well. On the other hand, mechanical weeding in organic crop rows allows the emergence of some herbs during part of the growing season. As plant species richness has been shown to positively correlate with soil fungal diversity, this could explain the lower fungal richness, phylogenetic diversity, and altered community composition in the IPM orchards (Vukicevich et al., 2016). All investigated fungal diversity indices were higher in drive rows compared to crop rows, confirming the findings of Hendgen et al. (2018) in vineyards. The herbaceous drive rows act as a permanent vegetation cover where the rhizosphere delivers a suitable habitat for soil microbes (Berg and Smalla, 2009). The root exudates supply nutrient sources, such as organic carbon and amino acids, and anti-pathogenic compounds, as well as take part in plant-microorganism interactions through the production of signaling molecules, e.g., acting as chemoattractant to microbes or microbial growth promotors (Haichar et al., 2014). Additionally, frequent mowing and litter decomposition in the drive rows increase carbon inputs to the soil, acting as a nutrient source for microbes. Indeed, we found higher organic matter content in drive rows compared to their adjacent crop row (Supplementary Table S6).

General fungal richness aside, understanding the management response of key fungal functional groups within the soil microbiome is critical as they strongly impact soil functioning (Barrios, 2007; Lüneberg et al., 2019; Lekberg et al., 2021). Both AMF abundance and the prevalence of Glomeromycota in the indicator species analysis and as keystone taxa in the co-occurrence networks were higher in the IPM than in the organic orchards. This is in line with previous studies finding either lower (Di Giacinto et al., 2020) or similar AMF occurrences (Turrini et al., 2017) in organic compared to IPM orchards. Whereas the response of AMF to herbicide applications has been reported to be highly variable, AMF may negatively respond to mechanical weeding in organic crop rows, likely explaining the increased occurrence of AMF in IPM orchards (Turrini et al., 2017; Mandl et al., 2018; Hage-Ahmed et al., 2019). On the other hand, we found no management effects on the number of plant pathogen reads, aligning with recent work (Hannula et al., 2021). While AMF were more abundant in drive rows, pathogenic fungi were more abundant in crop rows. This is in line with other work that has shown that cover crop diversity increased AMF root colonization, likely due to the increased availability of root carbon sources, whereas it reduced the amount of soil-borne pathogens, possibly because of the antagonistic role of AMF (Haichar et al., 2014; Vukicevich et al., 2016; Bowles et al., 2017).

Our fungal networks were relatively similar in both management types and across locations in terms of complexity and connectivity, with a notable exception for the more complex and connected IPM crop row network. It is known that the complexity of microbial co-occurrence networks in the soil is reduced with increasing disturbance and compaction (Hartmann et al., 2014; Karimi et al., 2017; Xia et al., 2022). However, the soil in IPM crop rows is not mechanically disturbed, as opposed to organic crop rows, which are mechanically weeded, or drive rows, which are frequently compacted by heavy machinery. The increased complexity of our IPM crop row network could thus be attributed to the relatively undisturbed soil under IPM crop rows. This is contrary to its relatively low observed fungal richness and phylogenetic diversity, highlighting the limited usefulness of simple diversity indices when quantifying management impacts (Hartmann et al., 2015; Karimi et al., 2017).

### Management and Location Effects on Soil Bacteria

Unlike fungi, orchard management did not affect bacterial diversity indices, nor bacterial community composition. This lack of management effects has previously also been observed for bacterial diversity indices in coffee plantations (Jurburg et al., 2020) and for bacterial community composition in vineyards (Hendgen et al., 2018). Bacteria have indeed been found to be less sensitive to land use, as they respond more directly to soil properties (Petersen et al., 2019). As soil characteristics were mostly accounted for in our analyses through integrating them in the models as covariates, this might explain the lack of a management effect on bacterial diversity and community composition. It is for instance known that soil bacterial diversity and community composition are heavily influenced by soil pH levels and organic matter content, as observed here (Fierer and Jackson, 2006; Rousk et al., 2010; Griffiths et al., 2011; Yashiro et al., 2016; Karimi et al., 2017). In addition, the spatial autocorrelation identified here has also been detected in other systems, indicating the importance of spatial processes in the distribution of bacterial communities (Constancias et al., 2015; Shi et al., 2015). Fungi are also known to be more sensitive to plant diversity loss than bacteria due to their often more specific interactions with plant species, both through root exudates and litter inputs (Eisenhauer et al., 2017; Jurburg et al., 2020). On the other hand, bacterial diversity and phylogenetic diversity were higher in crop rows, which also harbored distinct bacterial communities compared to drive rows. Bacteria have a relatively high resistance to herbicides and physical soil disturbances, which could have led to the higher diversity indices found in crop rows (Schlatter et al., 2017; Chou et al., 2018; Mandl et al., 2018).

The bacterial co-occurrence networks were generally relatively similar across both management types, which were also found in an arable long-term trial cultivating wheat and rapeseed (Finn et al., 2021). However, the organic drive row network was more complex and contained more keystone taxa than the other orchard networks. The increased complexity in the organic drive rows might be due to the increased plant species diversity observed in its permanent herbaceous vegetation crop (Vukicevich et al., 2016). Additionally, bacterial networks have been found to have an increased number of positive links under a cover crop, indicating decreased competition and increased cooperation, potentially due to greater niche separation (Zheng et al., 2018). Again, we observed a disparity between the diversity indices responses and the network analyses. Whereas nutrient additions and soil disturbances in crop rows homogenize microhabitats in the soil, potentially resulting in less complex networks, they increased the overall nutrient levels, possibly leading to higher diversity indices (Gossner et al., 2016; Szoboszlay et al., 2017).

### Land Use Effects on the Soil Microbiome

When comparing the effects of orchard management on microbial diversity indices in the drive rows to the semi-natural grassland benchmark, the latter had a lower fungal OTU richness and a lower value for all bacterial diversity indices compared to both orchard types. There was also a clear difference in the fungal community composition among all three land use types, though there was a higher similarity between both orchard systems. Additionally, bacterial community composition did not differ between orchard types, while there was a clear separation from the grassland communities. Other studies have reported both an increased fungal and bacterial diversity and a community shift in agricultural systems compared to more natural systems (Szoboszlay et al., 2017; Terrat et al., 2017; George et al., 2019; Petersen et al., 2019; Lammel et al., 2021). This has been attributed to the higher fertilizer inputs, increased soil compaction, and more frequent soil disturbance transferring nutrients into the soil and increasing microbial diversity indices (Hartmann et al., 2014; Szoboszlay et al., 2017; Longepierre et al., 2021). Additionally, disturbance might induce dormancy in some microbial taxa, or reduce dominant OTUs, opening opportunities for a range of weaker competitors and thus increasing diversity (Wang et al., 2014; Szoboszlay et al., 2017; George et al., 2019). These land use effects were more apparent for bacteria compared to fungi, which is in line with similar findings in tropical agricultural systems (Petersen et al., 2019; Lammel et al., 2021).

We found functionally enhanced communities in semi-natural grasslands compared to the herbaceous drive rows in both orchard types. The higher saprotroph read counts found in the semi-natural grasslands compared to the drive rows in both orchard types might be attributed to the much higher plant diversity found in these semi-natural grasslands, which changes the quality and diversity of litter inputs to the soil, affecting these primary decomposers (Millard and Singh, 2010; Francioli et al., 2021). Surprisingly, the number of AMF reads in organic drive rows was lower than in IPM drive rows, while we found no difference in AMF abundance between IPM and the benchmark grasslands. This is in contrast to reduced AMF abundances found under synthetic fertilization compared to a non-fertilized control and reduced AMF richness in grasslands compared to arable systems (Hannula et al., 2021; Zhu et al., 2021). Instead, other factors may influence AMF abundance in drive rows. It has for instance been suggested that compaction is higher in organic drive rows, potentially due to increased traffic as a result of higher labor inputs and the frequent need for fungicide applications under wet conditions, which may reduce mycorrhizal formation (Entry et al., 2002; Vogeler et al., 2006).

Both the fungal and bacterial semi-natural grassland co-occurrence networks were distinctly more complex, more connected and contained more keystone taxa than any of the orchard networks. In addition, the bacterial grassland network contained nodes from a much wider range of phyla in comparison with orchard networks. The increased abundance and diversity of keystone taxa in the semi-natural grassland networks further amplified network complexity (Herren and McMahon, 2018). As we only included positive correlations, links between network nodes are associated with potential co-dependent relationships, such as biotic interactions and niche sharing (Karimi et al., 2017; Xue et al., 2022). The tightened positive interactions found in both grassland networks could be related to improved trophic interactions and cooperation, as increased nutrient levels may displace the need for robust trophic interactions between different microbial functional groups (Holtkamp et al., 2011; Wu et al., 2021). Additionally, network complexity in orchards might be reduced by increased disturbance, compaction, and fertilization due to the homogenization of available soil niches (Fraterrigo et al., 2005).

Agricultural management substantially affects soil microbiomes and the effects of conventional and organic management have frequently been evaluated. However, to adequately quantify the importance of these effects, it is important to include a (semi-)natural benchmark. This study found effects of both IPM and organic agricultural management on the soil microbiome in the crop and drive rows of apple orchards. Our results indicate the importance of management practices that minimize both soil disturbance and compaction and favor a more species-rich permanent vegetation cover for maintaining more complex and connected microbiomes in perennial agricultural systems. However, the agricultural soil microbiomes in the drive rows were no match for the complexity and connectedness found in the semi-natural benchmark, demonstrating that even more nature-friendly agricultural management practices strongly affect the soil microbiome and highlighting the need for the conservation of (semi-)natural systems to harbor robust and functionally diverse fungal and bacterial communities.

## Data Availability Statement

The datasets presented in this study can be found in online repositories. The names of the repository/repositories and accession number(s) can be found at: https://www.ncbi.nlm.nih.gov/, PRJNA786545.

## Author Contributions

EH: conceptualization and design, field work, data analysis and interpretation, drafting the manuscript, revision, and editing. GP: laboratory analysis, manuscript revision, and editing. OH: conceptualization and design, manuscript revision and editing, and supervising the research. All authors contributed to the article and approved the submitted version.

## Funding

This project was funded by Research Foundation—Flanders (71635) and by the KU Leuven Special Research Fund (BOF; project C24/18/034).

## Conflict of Interest

The authors declare that the research was conducted in the absence of any commercial or financial relationships that could be construed as a potential conflict of interest.

## Publisher’s Note

All claims expressed in this article are solely those of the authors and do not necessarily represent those of their affiliated organizations, or those of the publisher, the editors and the reviewers. Any product that may be evaluated in this article, or claim that may be made by its manufacturer, is not guaranteed or endorsed by the publisher.
