# Supplementary material for: Soil Microbiomes in Apple Orchards Are Influenced by the Type of Agricultural Management but Never Match the Complexity and Connectivity of a Semi-natural Benchmark
Source: Front Microbiol. 2022 Feb 16;13:830668. doi: 10.3389/fmicb.2022.830668 (PMC8888915; doi:10.3389/fmicb.2022.830668)
Supplement: Supplementary file 1 [file Data_Sheet_1.docx]

Supplementary Material

# Supplementary Figures and Tables

## Supplementary Tables

**Table S1:** Results of the (generalized) linear mixed models for Hill numbers of order 0 (OTU richness) and order 1 (^1^D) and phylogenetic diversity (PD). Models were constructed for all orchard data, excluding the semi-natural reference, and orchard drive row data with the semi-natural reference separately. Test statistics (χ^2^), degrees of freedom (Df) and p-values (P) are given for each variable selected in the model selection procedure.

|  |  | Orchards | | | | | | Semi-natural grasslands as reference | | | | | |
| --- | --- | --- | --- | --- | --- | --- | --- | --- | --- | --- | --- | --- | --- |
|  |  | Fungi | | | Bacteria | | | Fungi | | | Bacteria | | |
|  | Selected variables | χ^2^ | Df | P | χ^2^ | Df | P | χ^2^ | Df | P | χ^2^ | Df | P |
| OTU richness | Land use | 12.53 | 1 | 0.0004 |  |  |  | 8.42 | 2 | 0.015 | 21.24 | 2 | <0.0001 |
|  | Location | 58.54 | 1 | <0.0001 |  |  |  |  |  |  |  |  |  |
|  | Land use:Loc | 38.68 | 1 | <0.0001 |  |  |  |  |  |  |  |  |  |
|  | P | 19.99 | 1 | <0.0001 | 78.86 | 1 | <0.0001 |  |  |  | 13.25 | 1 | 0.00027 |
|  | Water content | 17.22 | 1 | <0.0001 | 188.90 | 1 | <0.0001 | 9.94 | 1 | 0.0016 | 24.72 | 1 | <0.0001 |
|  | Organic matter | 179.81 | 1 | <0.0001 |  |  |  | 8.12 | 1 | 0.0043 |  |  |  |
|  | pH |  |  |  | 14.64 | 1 | 0.00013 | 26.29 | 1 | <0.0001 | 29.42 | 1 | <0.0001 |
|  | NO3 |  |  |  |  |  |  |  |  |  | 6.70 | 1 | 0.0097 |
|  | NH4 | 49.74 | 1 | <0.0001 |  |  |  | 33.94 | 1 | <0.0001 | 53.68 | 1 | <0.0001 |
|  | MEM4 |  |  |  | 17.36 | 1 | <0.0001 |  |  |  |  |  |  |
|  | MEM17 |  |  |  | 13.98 | 1 | 0.00018 |  |  |  |  |  |  |
| ^1^D | Land use |  |  |  |  |  |  |  |  |  | 16.40 | 2 | 0.00027 |
|  | Location | 19.70 | 1 | <0.0001 | 92.83 | 1 | <0.0001 |  |  |  |  |  |  |
|  | MEM3 | 17.31 | 1 | <0.0001 |  |  |  |  |  |  |  |  |  |
|  | MEM4 |  |  |  | 8.60 | 1 | 0.0034 |  |  |  |  |  |  |
|  | MEM5 |  |  |  | 7.94 | 1 | 0.0048 |  |  |  |  |  |  |
|  | MEM6 | 10.19 | 1 | 0.0014 | 16.17 | 1 | <0.0001 |  |  |  | 29.42 | 1 | 0.0033 |
|  | MEM7 |  |  |  | 12.20 | 1 | 0.00048 |  |  |  |  |  |  |
|  | MEM8 | 15.30 | 1 | <0.0001 |  |  |  |  |  |  |  |  |  |
|  | MEM9 |  |  |  | 10.49 | 1 | 0.0012 |  |  |  |  |  |  |
|  | MEM12 | 6.75 | 1 | 0.0094 |  |  |  |  |  |  | 13.25 | 1 | 0.0071 |
|  | MEM17 |  |  |  | 7.86 | 1 | 0.0051 |  |  |  |  |  |  |
| PD | Land use | 6.95 | 1 | 0.0084 | *0.000* | *1* | *1.00* |  |  |  | 15.74 | 2 | 0.00038 |
|  | Location | 14.77 | 1 | 0.00012 | 22.81 | 1 | <0.0001 |  |  |  |  |  |  |
|  | Land use:Loc | 3.35 | 1 | 0.067 |  |  |  |  |  |  |  |  |  |
|  | Soil type | *0.20* | *3* | *0.98* |  |  |  |  |  |  |  |  |  |
|  | P | *1.24* | *1* | *0.26* |  |  |  |  |  |  |  |  |  |
|  | Water content | *1.24* | *1* | *0.27* |  |  |  | 8.85 | 1 | 0.0029 |  |  |  |
|  | Organic matter | 18.05 | 1 | <0.0001 |  |  |  | 8.63 | 1 | 0.0033 |  |  |  |
|  | pH | *1.80* | *1* | *0.18* |  |  |  |  |  |  |  |  |  |
|  | NO3 | *0.023* | *1* | *0.88* |  |  |  |  |  |  |  |  |  |
|  | NH4 | 10.39 | 1 | 0.0013 |  |  |  |  |  |  |  |  |  |
|  | MEM4 |  |  |  | 11.86 | 1 | 0.00057 |  |  |  |  |  |  |
|  | MEM17 |  |  |  | 8.28 | 1 | 0.0040 |  |  |  |  |  |  |

**Table S2:** Results of the canonical redundancy analyses (RDA) on fungal and bacterial community composition. RDAs were performed for all orchard data, excluding the semi-natural reference, and orchard drive row data with the semi-natural reference separately. Adjusted R^2^, test statistics (F), explained variance (var), degrees of freedom (Df) and p-values are given for all full models. Test statistics (F), explained variance (var), degrees of freedom (Df) and p-values are also given for each variable selected in the model selection procedure.

|  | Orchards | | | | | | | | Semi-natural grasslands as reference | | | | | | | |
| --- | --- | --- | --- | --- | --- | --- | --- | --- | --- | --- | --- | --- | --- | --- | --- | --- |
|  | Fungi | | | | Bacteria | | | | Fungi | | | | Bacteria | | | |
| Adjusted R^2^ | 0.17 | | | | 0.16 | | | | 0.16 | | | | 0.30 | | | |
|  | F | var | Df | P | F | var | Df | P | F | var | Df | P | F | var | Df | P |
| Full model | 6.36 | 0.11 | 12 | 0.001 | 7.91 | 0.046 | 9 | 0.001 | 6.88 | 0.12 | 8 | 0.001 | 9.53 | 0.11 | 12 | 0.001 |
| Land use | 24.44 | 0.034 | 1 | 0.001 |  |  |  |  | 12.43 | 0.054 | 2 | 0.001 | 8.30 | 0.016 | 1 | 0.001 |
| Location | 13.41 | 0.019 | 1 | 0.001 | 20.71 | 0.014 | 1 | 0.001 |  |  |  |  |  |  |  |  |
| Land use:Loc | 4.51 | 0.0064 | 1 | 0.001 |  |  |  |  |  |  |  |  |  |  |  |  |
| P | 2.54 | 0.0036 | 1 | 0.001 | 7.61 | 0.0050 | 1 | 0.001 | 1.73 | 0.0037 | 1 | 0.01 | 3.31 | 0.0032 | 1 | 0.005 |
| Water content |  |  |  |  | 7.61 | 0.0050 | 1 | 0.001 | 2.76 | 0.0060 | 1 | 0.001 | 5.24 | 0.0051 | 1 | 0.001 |
| Organic matter | 3.95 | 0.0056 | 1 | 0.001 | 3.05 | 0.0020 | 1 | 0.002 |  |  |  |  | 4.97 | 0.0048 | 1 | 0.002 |
| pH | 4.99 | 0.0070 | 1 | 0.001 | 10.65 | 0.0069 | 1 | 0.001 | 19.38 | 0.042 | 1 | 0.001 | 65.36 | 0.064 | 1 | 0.001 |
| NO3 |  |  |  |  |  |  |  |  |  |  |  |  | 1.10 | 0.0011 | 1 | 0.275 |
| MEM1 | 4.12 | 0.0058 | 1 | 0.001 |  |  |  |  |  |  |  |  |  |  |  |  |
| MEM4 | 5.66 | 0.0080 | 1 | 0.001 | 9.70 | 0.0063 | 1 | 0.001 |  |  |  |  | 3.82 | 0.0037 | 1 | 0.003 |
| MEM6 |  |  |  |  | 5.64 | 0.0037 | 1 | 0.001 |  |  |  |  | 4.03 | 0.0039 | 1 | 0.003 |
| MEM7 | 3.40 | 0.0048 | 1 | 0.001 | 3.70 | 0.0024 | 1 | 0.001 |  |  |  |  |  |  |  |  |
| MEM9 | 4.49 | 0.0063 | 1 | 0.001 |  |  |  |  | 2.16 | 0.0047 | 1 | 0.001 |  |  |  |  |
| MEM10 |  |  |  |  |  |  |  |  |  |  |  |  | 2.02 | 0.0020 | 1 | 0.029 |
| MEM12 |  |  |  |  | 2.56 | 0.0017 | 1 | 0.001 | 2.25 | 0.0049 | 1 | 0.001 | 6.20 | 0.0060 | 1 | 0.001 |
| MEM14 | 2.15 | 0.0030 | 1 | 0.001 |  |  |  |  |  |  |  |  |  |  |  |  |
| MEM15 |  |  |  |  |  |  |  |  |  |  |  |  | 1.75 | 0.0017 | 1 | 0.069 |
| MEM16 | 2.71 | 0.0038 | 1 | 0.001 |  |  |  |  | 1.93 | 0.0042 | 1 | 0.005 |  |  |  |  |

**Table S3:** Results of the generalized linear mixed models for the read count of different fungal functional groups (saprotrophic fungi, AMF and plant pathogens). Models were constructed for all orchard data, excluding the semi-natural reference, and orchard drive row data with the semi-natural reference separately. Test statistics (χ^2^), degrees of freedom (Df) and p-values (P) are given for each selected variable after the model selection procedure.

|  | Orchards | | | | | | | | | Semi-natural grasslands as reference | | | | | | | | |
| --- | --- | --- | --- | --- | --- | --- | --- | --- | --- | --- | --- | --- | --- | --- | --- | --- | --- | --- |
|  | Saprotrophs | | | AMF | | | Plant pathogens | | | Saprotrophs | | | AMF | | | Plant pathogens | | |
|  | χ^2^ | Df | P | χ^2^ | Df | P | χ^2^ | Df | P | χ^2^ | Df | P | χ^2^ | Df | P | χ^2^ | Df | P |
| Land use |  |  |  | 4.00 | 1 | 0.045 | *0.0047* | *1* | *0.95* | 17.41 | 2 | 0.00017 | 7.69 | 2 | 0.024 | 8.71 | 2 | 0.013 |
| Location |  |  |  | 942.58 | 1 | <0.0001 | 1324.38 | 1 | <0.0001 |  |  |  |  |  |  |  |  |  |
| Land use:Loc |  |  |  | 379.97 | 1 | <0.0001 | 1863.91 | 1 | <0.0001 |  |  |  |  |  |  |  |  |  |
| P |  |  |  | 259.24 | 1 | <0.0001 | 764.04 | 1 | <0.0001 | 927.27 | 1 | <0.0001 | 5.09 | 1 | 0.024 | 14.05 | 1 | 0.00018 |
| Water content | 6.82 | 1 | 0.0090 | 36.02 | 1 | <0.0001 | 1208.53 | 1 | <0.0001 | 11.48 | 1 | 0.00071 | 106.98 | 1 | <0.0001 |  |  |  |
| Organic matter | 34.06 | 1 | <0.0001 |  |  |  | 359.68 | 1 | <0.0001 | 328.16 | 1 | <0.0001 | 12.53 | 1 | 0.00040 | 65.43 | 1 | <0.0001 |
| pH | 2080.84 | 1 | <0.0001 |  |  |  | 1586.32 | 1 | <0.0001 |  |  |  |  |  |  | 101.9 | 1 | <0.0001 |
| NO3 |  |  |  |  |  |  | 8.61 | 1 | <0.0001 | 1652.36 | 1 | <0.0001 | 15.07 | 1 | 0.00010 | 45.12 | 1 | <0.0001 |
| NH4 |  |  |  | 10.57 | 1 | 0.0012 | 61.71 | 1 | <0.0001 |  |  |  | 10.07 | 1 | 0.0015 | 1533.7 | 1 | <0.0001 |
| MEM4 |  |  |  | 10.39 | 1 | 0.0013 |  |  |  |  |  |  |  |  |  |  |  |  |
| MEM6 |  |  |  |  |  |  | 13.68 | 1 | 0.00022 |  |  |  |  |  |  |  |  |  |
| MEM7 |  |  |  |  |  |  | 26.45 | 1 | <0.0001 |  |  |  |  |  |  | 8.59 | 1 | 0.0034 |
| MEM17 |  |  |  |  |  |  |  |  |  | 6.52 | 1 | 0.01 |  |  |  |  |  |  |

**Table S4:** Results of the generalized linear mixed models for the read count of different bacterial functional groups. Models were constructed for all orchard data, excluding the semi-natural reference, and orchard drive row data with the semi-natural reference separately. Test statistics (χ^2^), degrees of freedom (Df) and p-values (P) are given for each selected variable after the model selection procedure.

|  | Orchards | | | | | | | | | | | | | Semi-natural grasslands as reference | | | | | | | | | | | |
| --- | --- | --- | --- | --- | --- | --- | --- | --- | --- | --- | --- | --- | --- | --- | --- | --- | --- | --- | --- | --- | --- | --- | --- | --- | --- |
|  | Sulfate  reducer | | | | Ammonia  oxidizer | | | Dehalogenation | | | Xylan  degrader | | | Sulfate  reducer | | | Ammonia  oxidizer | | | Dehalogenation | | | Xylan  degrader | | |
|  | χ2 | Df | P | | χ2 | Df | P | χ2 | Df | P | χ2 | Df | P | χ2 | Df | P | χ2 | Df | P | χ2 | Df | P | χ2 | Df | P |
| Land use | 3.37 | 1 | | 0.066 |  |  |  |  |  |  |  |  |  |  |  |  | 55.55 | 2 | <0.0001 |  |  |  |  |  |  |
| Location |  |  | |  |  |  |  |  |  |  |  |  |  |  |  |  |  |  |  |  |  |  |  |  |  |
| Land use:Loc |  |  | |  |  |  |  |  |  |  |  |  |  |  |  |  |  |  |  |  |  |  |  |  |  |
| Soil type | 1.44 | 3 | 0.70 | |  |  |  |  |  |  |  |  |  |  |  |  |  |  |  |  |  |  |  |  |  |
| P |  |  | |  |  |  |  |  |  |  |  |  |  | 108.72 | 1 | <0.0001 | 75.57 | 1 | <0.0001 | 23.85 | 1 | <0.0001 | 45.61 | 1 | <0.0001 |
| Water content |  |  |  | |  |  |  |  |  |  |  |  |  |  |  |  | 136.05 | 1 | <0.0001 | 25.00 | 1 | <0.0001 | 31.22 | 1 | <0.0001 |
| Organic matter |  |  |  | |  |  |  |  |  |  |  |  |  |  |  |  |  |  |  |  |  |  |  |  |  |
| pH |  |  |  | |  |  |  |  |  |  |  |  |  | 16.24 | 1 | <0.0001 |  |  |  |  |  |  | 49.10 | 1 | <0.0001 |
| MEM2 | 20.32 | 1 | | <0.0001 |  |  |  | 17.10 | 1 | <0.0001 |  |  |  | 27.41 | 1 | <0.0001 |  |  |  | 26.37 | 1 | <0.0001 | 18.40 | 1 | <0.0001 |
| MEM4 | 9.03 | 1 | 0.015 | |  |  |  | 5.56 | 1 | 0.018 |  |  |  |  |  |  |  |  |  |  |  |  |  |  |  |
| MEM6 | 7.47 | 1 | 0.0027 | | 5.36 | 1 | 0.021 | 10.13 | 1 | 0.0015 |  |  |  |  |  |  |  |  |  |  |  |  |  |  |  |
| MEM7 | 6.01 | 1 | 0.014 | |  |  |  | 8.30 | 1 | 0.0040 |  |  |  |  |  |  |  |  |  |  |  |  |  |  |  |
| MEM10 | 1.12 | 1 | 0.29 | |  |  |  |  |  |  |  |  |  |  |  |  | 5.54 | 1 | 0.019 |  |  |  |  |  |  |
| MEM11 |  |  |  | |  |  |  |  |  |  | 10.81 | 1 | 0.0010 |  |  |  |  |  |  |  |  |  |  |  |  |
| MEM12 | 1.83 | 1 | 0.18 | |  |  |  |  |  |  |  |  |  |  |  |  |  |  |  |  |  |  |  |  |  |

**Table S5:** Topology parameters associated with the constructed co-occurrence networks for each land use type.

|  | Fungi | | | | | Bacteria | | | | |
| --- | --- | --- | --- | --- | --- | --- | --- | --- | --- | --- |
|  | IPM  crop row | IPM drive row | Organic crop row | Organic drive row | Semi-natural  grassland | IPM  crop row | IPM drive row | Organic crop row | Organic drive row | Semi-natural  grassland |
| No. nodes | 110 | 67 | 47 | 59 | 286 | 619 | 596 | 614 | 708 | 1164 |
| No. edges | 155 | 47 | 38 | 50 | 499 | 2222 | 2914 | 2660 | 7208 | 43628 |
| Diameter | 10 | 5 | 3 | 4 | 17 | 11 | 16 | 14 | 13 | 10 |
| Degree | 0.11 | 0.024 | 0.074 | 0.057 | 0.094 | 0.064 | 0.15 | 0.084 | 0.17 | 0.17 |
| Closeness centrality | 0.0060 | 0.0019 | 0.0031 | 0.0040 | 0.0028 | 0.0052 | 0.0062 | 0.0060 | 0.016 | 0.17 |
| Betweenness centrality | 0.064 | 0.0035 | 0.0084 | 0.0084 | 0.055 | 0.11 | 0.16 | 0.17 | 0.26 | 0.047 |
| Average path length | 3.94 | 1.58 | 1.63 | 1.76 | 5.28 | 5.16 | 5.51 | 5.60 | 4.93 | 3.51 |
| Modularity | 0.70 | 0.94 | 0.86 | 0.87 | 0.62 | 0.71 | 0.39 | 0.64 | 0.52 | 0.54 |

**Table S6:** Mean and standard deviation of soil variables for each land use type.

|  | IPM  crop row | IPM drive row | Organic crop row | Organic drive row | Semi-natural  grassland |
| --- | --- | --- | --- | --- | --- |
| pH | 7.42 ± 0.26 | 7.46 ± 0.31 | 7.27 ± 0.32 | 7.25 ± 0.33 | 6.59 ± 0.75 |
| Water content | 0.095 ± 0.022 | 0.12 ± 0.028 | 0.096 ± 0.028 | 0.13 ± 0.036 | 0.14 ± 0.067 |
| Organic matter content | 0.045 ± 0.0091 | 0.065 ± 0.014 | 0.052 ± 0.0093 | 0.065 ± 0.017 | 0.068 ± 0.027 |
| P (mg/kg soil) | 2.43 ± 3.73 | 0.92 ± 1.08 | 2.36 ± 3.39 | 0.91 ± 1.46 | 1.38 ± 1.50 |
| NO_3_^-^ (mg/kg soil) | 12.23 ± 10.63 | 15.82 ± 18.31 | 12.44 ± 13.10 | 14.33 ± 11.53 | 7.45 ± 8.28 |
| NH_4_ (mg/kg soil) | 2.30 ± 6.23 | 1.06 ± 1.30 | 4.10 ± 30.03 | 2.44 ± 10.84 | 3.85 ± 10.83 |

## Supplementary Figures

**
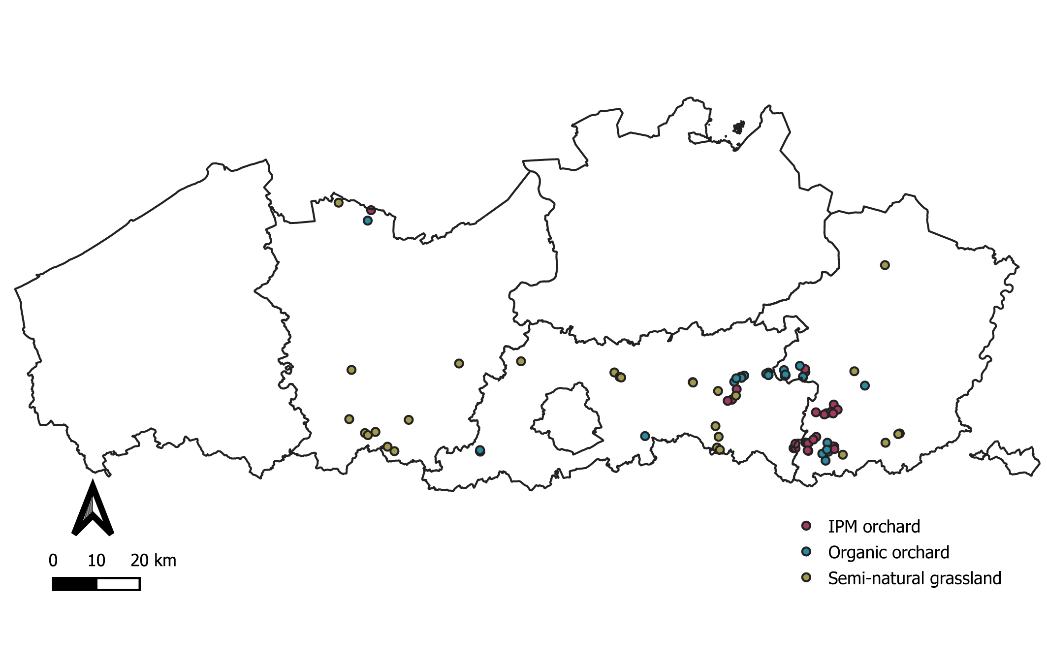
**

**Supplementary Figure S1: Map of the study area.** Locations of IPM and organic orchards and semi-natural grasslands used as study sites in Flanders, Belgium.

**
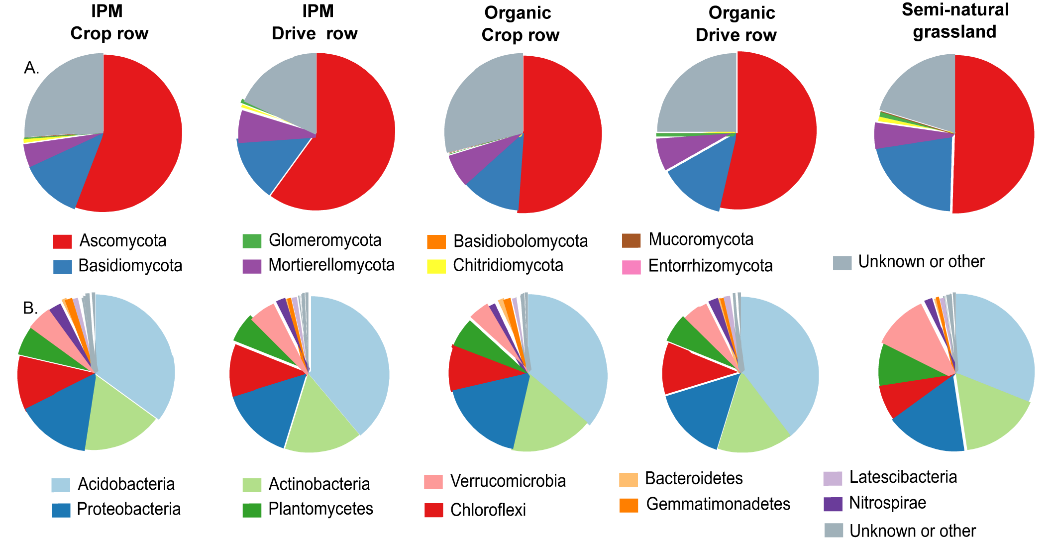
**

**Supplementary Figure S2: Proportional distribution of phyla across land use types.** Pie charts displaying the proportion of the most abundant phyla (> 1%) for fungal (A) and bacterial (B) communities in IPM and organic crop rows and drive rows with a semi-natural grassland reference.

**
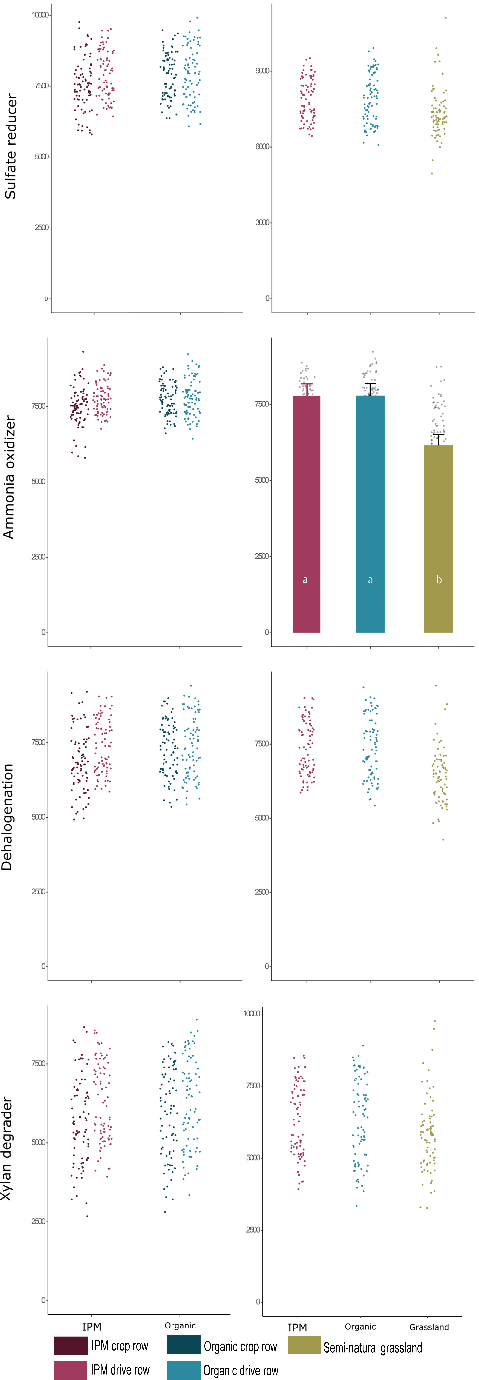
**

**Supplementary Figure S3: Read count of bacterial functional groups in IPM and organic orchards with a semi-natural grassland reference.** Modelled mean values ± 1SE and raw data points of sulfate reducer, ammonia oxidizer, dehalogenation and xylan degrader read counts across land use types. When land use or location were not selected during the model selection procedure, only raw data points are shown. Left panels: Crop rows (dark) and drive rows (light) of IPM and organic orchards. Right panels: Drive rows of IPM and organic orchards with a semi-natural grassland reference. Same lowercase letter indicates no statistically significant (P < 0.05) difference between land uses.
